# Use of the novel hemostatic textile Stasilon^® ^to arrest refractory retroperitoneal hemorrhage: a case report

**DOI:** 10.1186/1752-1947-4-20

**Published:** 2010-01-22

**Authors:** Preston B Rich, Christelle Douillet, Valorie Buchholz, David W Overby, Samuel W Jones, Bruce A Cairns

**Affiliations:** 1 Department of Surgery, Division of Trauma and Critical Care, University of North Carolina, Chapel Hill, NC 27599-7228, USA

## Abstract

**Introduction:**

Stasilon^® ^is a novel hemostatic woven textile composed of allergen-free fibers of continuous filament fiberglass and bamboo yarn. The development of this product resulted from controlled *in vitro *thrombogenic analysis of an array of potentially hemostatic textile materials and it has been cleared for both external and internal use by the United States Food and Drug Administration for the arrest of hemorrhage. The goal of the study was to assess the hemostatic and adhesive properties of Stasilon^® ^in the setting of life-threatening refractory hemorrhage.

**Case presentation:**

A 39-year-old Caucasian man presented with severe necrotic pancreatitis that failed multiple aggressive attempts to control associated bleeding with electrocautery, suture ligation, and sequential anatomic packing with cotton-based sponges. Subsequent retroperitoneal packing with Stasilon^® ^produced a non-adherent wound-dressing interface and resulted in the achievement of persistent hemostasis in the operative field.

**Conclusion:**

In our patient, Stasilon^® ^was demonstrated to be effective in the arrest of refractory hemorrhage.

## Introduction

Uncontrolled hemorrhage is a major contributor to both trauma-associated and intra-operative morbidity and mortality [1,2]. Achieving hemostasis is crucial in avoiding distributive shock and interrupting the progressive physiologic compromise that is often marked by dilutional coagulopathy, metabolic acidemia, and the sequelae of microcirculatory malperfusion [3,4]. Cotton-fiber-based dressings have traditionally been used liberally in wound management, but the minimally thrombogenic nature of their blood-matrix interface, the inherently adhesive nature of their surfaces to the wound bed, and their highly absorptive qualities make them less than ideal dressings. Modern textile sciences have enabled the development of novel hemostatic materials that have been specifically engineered to incorporate many of the most desirable qualities of the ideal dressing [5,6]. Stasilon^® ^is a novel hemostatic woven textile composed of allergen-free fibers of continuous filament fiberglass and bamboo yarn. The goal of this report is to document our experience with the use of Stasilon^® ^in a case of persistent retroperitoneal hemorrhage that could not be controlled by traditional means of securing hemostasis.

## Case presentation

A 39-year-old Caucasian man presented to our emergency department with a 1-day history of nausea, vomiting, and severe peri-umbilical pain that radiated to the mid-scapular region of his back. The patient had a history of muscular dystrophy, hypertension, and transient renal insufficiency. There was no elicited history of jaundice, cholelithiasis, or bleeding diathesis. The patient used smokeless tobacco products and consumed three or more alcoholic drinks per day. On admission, laboratory results revealed no leukocytosis, a normal serum hemoglobin concentration, and normal renal function. Serum lipase was elevated at 1985 U/l. Transaminases and alkaline phosphate levels were mildly elevated; serum bilirubin was normal. Abdominal ultrasonography demonstrated edema of the pancreatic head without evidence of cholelithiasis. Computed tomographic (CT) imaging with intravenous and oral contrast confirmed inhomogeneous pancreatic enhancement localized to the head and uncinate process without evidence of necrosis or associated hemorrhage. The patient was maintained nil by mouth and admitted to the hospital for intravenous resuscitation and monitoring.

The patient developed multiple system organ dysfunction secondary to pancreatitis. On day 28, CT scanning and percutaneous sampling confirmed infected pancreatic necrosis (>50%).

The patient was taken to the operating room for laparotomy and exploration of the retroperitoneum. Diffuse fibrinous inflammation of the intra-abdominal contents and omentum was identified as was extensive retroperitoneal necrosis of the pancreas and surrounding peri-pancreatic tissues. An 80% pancreatic necrosectomy was performed which was accompanied by significant retroperitoneal bleeding. Attempts at hemorrhage control with electrocautery and suture ligation were unsuccessful. Associated hypotension required damage control treatment [7]; the retroperitoneum was packed with cotton sponges, the abdomen left open under sterile dressings, and the patient was transferred to the intensive care unit (ICU).

Progressive coagulopathy and hemodynamic compromise necessitated continued transfusion of blood and blood products and the intravenous administration of activated Factor VII (VIIa). Several hours after ICU admission, the abdomen was re-explored at the bedside for unabated bleeding and continued hypotension, despite correction of coagulopathy. Two liters of hemoperitoneum were evacuated and diffuse microvascular bleeding was encountered in the retroperitoneum of the left upper quadrant. Electrocautery and suture ligation were ineffective and the abdomen was repacked with cotton laparotomy pads and the abdomen left open.

Mesenteric angiography was performed after failure to achieve operative hemostasis; this demonstrated splenic arterial thrombosis without active arterial bleeding. Hemodynamics briefly improved and the patient was returned to the operating room for re-exploration and pack removal. The cotton packs were noted to be densely adherent to the retroperitoneum and diffuse re-bleeding occurred after their removal. Cautery and suture ligation again yielded incomplete hemostasis and fresh packs were replaced. Following additional transfusion and resuscitation in the ICU, the patient was returned to the operating room for another attempt at pack removal. Again, hemodynamically significant bleeding occurred from the exposed retroperitoneum upon pack removal. A splenectomy was performed to reduce the potential for collateral bleeding in the setting of proximal splenic arterial thrombosis but significant bleeding continued from the pancreatic bed.

**Figure 1:**
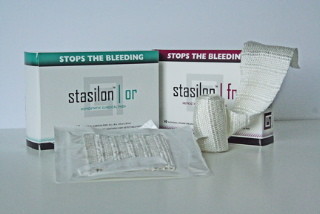
**Stasilon^®^**. Stasilon^® ^is a novel textile dressing composed of fiberglass and bamboo yarns incorporated into a proprietary weave. It has been cleared by the United States Food and Drug Administration for external and internal use and has been granted over-the-counter status.

**Figure 2:**
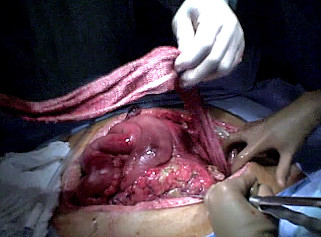
**Stasilon^® ^was non-adherent to the wound bed**. Forty-eight hours after open transabdominal packing, Stasilon^® ^rolls were noted to be non-adherent to the hemostatic wound bed and were easily removed atraumatically from the retroperitoneum.

**Figure 3:**
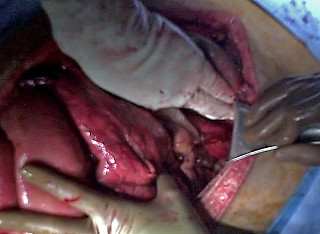
**After Stasilon^® ^removal, the retroperitoneum remained dry**. After pack removal, the retroperitoneum remained dry. The area was widely drained and the abdomen was able to be closed with Dexon™ mesh without plans for subsequent laparotomy.

Two 4-inch by 48-inch rolls of woven Stasilon^® ^textile (Entegrion, Research Triangle Park, NC, USA) were packed into the retroperitoneal space and pressure was applied for 4 minutes (Figure 1). Hemostasis was achieved with this maneuver, hemodynamics improved, and the patient was returned to the ICU for continued resuscitation with the new packs in place. Following the Stasilon^® ^packing, additional transfusions were not required, and hemodynamics were maintained. Forty-eight hours later, the patient was again returned to the operating room for re-exploration. At laparotomy, the packs were noted to be within a hemostatic field. Although directly contiguous with exposed structures, the Stasilon^® ^dressings did not adhere to the retroperitoneal tissues and the material was able to be removed from the pancreatic bed atraumatically (Figure 2). Complete hemostasis was noted in the retroperitoneum and left upper quadrant; no further hemostatic maneuvers were required (Figure 3). The region was widely drained and the abdomen closed with Dexon™ mesh.

Postoperatively, adequate hemodynamics were restored, resuscitation was successful, and vasopressor infusions were discontinued. Despite the eventual achievement of retroperitoneal hemostasis following the necrosectomy, the patient's subsequent hospital course was marked by progressive multiple system organ dysfunction and numerous associated complications. Ultimately, supportive interventions were ended and the patient died approximately 4 weeks after the final laparotomy that established retroperitoneal hemorrhage control.

## Discussion

The two component fibers used in the manufacture of Stasilon^® ^were selected from a panel of candidate materials based on their selective thrombogenicity as measured by acceleration of platelet-dependent turnover within the coagulation cascade and subsequent generation of thrombin. The resultant textile is generated from a proprietary weave of its two components, continuous filament type E glass (65%) and regenerated bamboo (35%). The weave pattern is unique and was engineered to optimize the contact surface area between the textile structure and blood components. The Stasilon^® ^fabric can be woven into widths of 1 to 4 inches from continuous filament fiberglass and bamboo precursors but is typically finished as a flat, 4-inch square (4 × 4 inches), single-layer pad that is individually packaged, sealed, depyrogenated, and sterilized with ethylene oxide gas. The manufacturing process can be modified to allow for variations in the dressing length and width including the packaging of sterile rolls of the product.

The process of Stasilon^® ^application is similar to that of familiar cotton products: sterile dressings are removed from the packaging, placed directly on the wound surface, and pressure is applied. Pre-clinical studies suggest that Stasilon^® ^may offer several clinical advantages over more traditional dressings including the induction of more rapid hemostasis, the corollary absorption of less shed blood, and less associated wound adherence resulting in more stable clot integrity proximate to the wound surface. Stasilon^® ^has been cleared by the United States Food and Drug Administration (USFDA) for external and internal use and has been granted over-the-counter status making it available without a prescription. Although Stasilon^® ^has been cleared for up to 30 days of implantation, we recommend its removal as soon as clinically feasible.

In this observational case study, we report the first intra-operative use of Stasilon^® ^as a hemostatic device to curtail uncontrolled hemorrhage from a surgical wound bed. Our patient suffered multiple episodes of life-threatening retroperitoneal exsanguination that were refractory to therapeutic interventions including liberal use of electrocautery and suture ligation, aggressive correction of associated coagulopathy and thrombocytopenia, sequential attempts at cotton-based packing, visceral angiography, and ultimately splenectomy. A single application of Stasilon^® ^packing in our patient resulted in complete resolution of associated hemorrhage, the attainment of hemodynamic stability with the ability to discontinue vasopressor support, and obviated the need for further perioperative transfusion of blood and blood products.

On re-exploration for pack removal 48 hours after placement, the Stasilon^® ^rolls were noted to be in direct juxtaposition to the hemostatic retroperitoneal surfaces but were not adherent to the surrounding tissue structures. This property facilitated atraumatic removal of the dressings and left behind stable thrombus on the wound bed. Although the patient ultimately died from multiple system organ dysfunction and complications resulting from severe underlying disease pathophysiology, we believe that the hemostatic properties and reduced tissue adhesion of the novel textile Stasilon^® ^directly contributed to the arrest of the life-threatening retroperitoneal hemorrhage encountered early in the patient's hospital course. Controlled studies of this novel dressing will facilitate objective analysis of its effectiveness.

## Conclusions

The use of Stasilon^® ^textile dressings in this patient produced robust hemostasis without associated wound adherence and resulted in the successful treatment of refractory hemorrhage. This novel hemostatic textile offers potential clinical advantages for the arrest of bleeding.

## Abbreviations

CT: computed tomography; ICU: intensive care unit; MRCP: magnetic resonance cholangiopancreatography; USFDA: United States Food and Drug Administration.

## Consent

Written informed consent was obtained from the patient's family for publication of this case report and any accompanying images. A copy of the written consent is available for review by the Editor-in-Chief of this journal.

## Competing interests

At the time of manuscript submission, all authors reported no conflict of interest. Dr. P. Rich currently serves as Chief Medical Officer for Entegrion. All other authors declare they have no competing interest.

## Authors' contributions

PR participated in the study design, the surgery, and manuscript preparation. CD participated in the study design and manuscript preparation, VB, DO, SJ, and BC participated in the study design and surgery. All authors read and approved the final manuscript.
